# Evaluation of neutrophil extracellular traps as the circulating marker for patients with acute coronary syndrome and acute ischemic stroke

**DOI:** 10.1002/jcla.23190

**Published:** 2020-01-06

**Authors:** Hyeon‐Ho Lim, In‐Hwa Jeong, Gyu‐Dae An, Kwang‐Sook Woo, Kyeong‐Hee Kim, Jeong‐Man Kim, Seong‐Hoon Yun, Joo‐In Park, Jae‐Kwan Cha, Moo‐Hyun Kim, Jin‐Yeong Han

**Affiliations:** ^1^ Department of Laboratory Medicine Dong‐A University College of Medicine Busan Korea; ^2^ Department of Biochemistry Dong‐A University College of Medicine Busan Korea; ^3^ Department of Neurology Dong‐A University College of Medicine Busan Korea; ^4^ Department of Cardiology Dong‐A University College of Medicine Busan Korea

**Keywords:** acute coronary syndrome, acute ischemic stroke, circulating marker, neutrophil extracellular traps, platelet activation

## Abstract

**Introduction:**

Neutrophil extracellular traps (NETs) are known to be induced by various factors. In this study, we tried to identify circulating levels of NETs in patients with acute coronary syndrome (ACS) and acute ischemic stroke (AIS) and to confirm its suitability as a new circulating marker in their detection.

**Methods:**

We prospectively enrolled 95 patients with a diagnosis of ACS (N = 37) or AIS (N = 58) in Dong‐A University Hospital, Busan, Korea. The control group was selected from healthy adults (N = 25) who visited the hospital for health screening. Circulating levels of NETs were evaluated by measuring plasma concentrations of double‐stranded DNA (dsDNA) and DNA‐histone complex.

**Results:**

The concentrations of dsDNA were statistically higher in patients with ACS or AIS than those in the control group (both *P* < .001). In the univariable and multivariable analyses, statistically significant risk factors were troponin I (TnI) level and dsDNA concentration in the ACS group (*P* = .046 and *P* = .015, respectively) and only dsDNA concentration in the AIS group (*P* = .002). In the receiver operating characteristic curve analyses, the area under the curve values for TnI level and dsDNA concentration in the ACS group were 0.878 and 0.968, respectively, and the value for dsDNA concentration in the AIS group was 0.859.

**Conclusions:**

In this study, it was confirmed that the circulating level of NETs was increased in patients with ACS and AIS at initial presentation. Findings in this study show that NETs could be used as a new circulating marker for the initial diagnosis of ACS or AIS.

## 1 INTRODUCTION

The number of Korean patients with acute coronary syndrome (ACS) and acute ischemic stroke (AIS) increases every year due to an aging population. As a result, patients with these diseases face a rapidly growing economic burden.^1^

As a common mechanism of ACS, the rupture of the atherosclerotic plaque causes partial or complete occlusion of the coronary artery. When the collagen of the endothelium is exposed by the collapse of the plaque, coagulation cascades start with platelet activation, leading to thrombus formation.^2^ In addition, it has been shown that excessive hyperactivity of platelets increases the risk of thromboembolism, leading to excessive formation of abnormal thrombosis together with atherosclerotic lesions, and is also a major factor in causing AIS.^3^ The interaction between platelets and vascular endothelial cells could induce local inflammatory conditions in the blood vessels, leading to microcirculatory disturbances that promote the gradual progression to atherosclerosis.^4^, ^5^, ^6^, ^7^

Meanwhile, the neutrophil is the most abundant cell type in the leukocyte and plays a crucial role in the innate immune system, which serves as the first line barrier against microorganisms.^8^, ^9^ In 2004, Brinkmann et al found that activated neutrophils released histones, protein granules, and double‐stranded DNA (dsDNA) to form fiber‐like structures, termed neutrophil extracellular traps (NETs).^10^ These NETs are known to be induced by various factors such as microorganisms, antibodies, activated platelets, and reactive oxygen species.^11^

Although NETs have been studied mainly in terms of host defense mechanisms, there has also been interest in NETs for pathogenetic aspects of certain noninfectious diseases. For instance, the involvement of NETs in thrombosis formation has been demonstrated.^12^ In addition to their immunological role, NETs provide a scaffold for red blood cells and platelets, contributing to coagulation cascades and formation of arterial and venous thromboses.^13^, ^14^ However, few analyses on NETs in patients with cardiocerebrovascular diseases exist. Therefore, there are limitations for their application to clinical fields.

In this study, we attempted to identify circulating levels of NETs using samples from patients with ACS and AIS and to confirm its suitability as a new circulating marker in detecting them. The circulating levels of NETs were evaluated by measuring concentrations of dsDNA. To clarify the conclusions, we performed an additional assay measuring the DNA‐histone complex, which was selected as the second marker. This selection was based on the observation that when neutrophils are activated by other stimuli, even in patients with ACS and AIS, they release DNA, which binds to histones and other cytoplasmic components.^10^ As well, the DNA‐histone complex is a relatively easy‐to‐measure test item in the laboratory. The results were analyzed in comparison with other clinical and laboratory markers.

## 2 MATERIALS AND METHODS

### 2.1 Study population and patient characteristics

Between October 2017 and August 2018, we prospectively enrolled 95 consecutive patients newly diagnosed with ACS (N = 37) or AIS (N = 58) in Dong‐A University Hospital, Busan, Korea. The diagnosis of ACS, defined as unstable angina (UA) (N = 21), non‐ST elevation myocardial infarction (NSTEMI) (N = 14), and ST elevation myocardial infarction (STEMI) (N = 2), was based on the patient's symptoms, physical examination, electrocardiogram, echocardiography, cardiac enzyme level, and other laboratory findings. Similarly, the diagnosis of AIS was based on the patient's symptoms, physical examination, Doppler ultrasonography, brain MRI, and brain CT findings.

With consideration of patient age and sex ratio, 25 adults who visited the hospital for health screening were selected as the control group. We used samples that showed normal results in complete blood count, blood coagulation test, liver function test, renal function test, urine test, fecal occult blood test, immunoserology test, and tumor marker test. The samples with out‐of‐reference values in the mentioned test items were excluded. Written consents from the patients were waived because it was a study using residual samples without any additional blood collection.

After their dsDNA concentrations were measured with the samples, statistical analyses were performed to compare the values with those of the control group and we investigated whether the values could be an informative marker at an initial stage of ACS and AIS. During the follow‐up period, we investigated occurrences of major adverse cardiac events (MACEs), such as myocardial infarction, restenosis, cardiac death, ischemic stroke, and so on.

Clinical and laboratory data were collected by a review of patients’ medical records. The Institutional Review Board of Dong‐A University Hospital approved this study.

### 2.2 Blood samples and assays

Blood samples were collected in sodium citrate tubes. Within 1 hour after collection, the blood samples were centrifuged at 1600 × g for 15 minutes. The prothrombin time (PT) and activated partial thromboplastin time (aPTT) were tested using a CS‐5100 coagulation analyzer (Sysmex). After these laboratory tests, leftover plasma samples with 1800 μL or more were selected for this study and stored at −80°C until measurement of their dsDNA concentrations. The stored samples were referred to Seegene medical foundation. The dsDNA concentrations were measured with Quant‐iT™ PicoGreen^®^ dsDNA reagent and kit (Molecular Probes) by which the dsDNA was fluorescently stained. The Synergy™ H1m Hybrid Multi‐Mode Microplate Reader (BioTek) was used for quantification of the stained dsDNA, and its filter setting was excitation/emission of 485 nm/528 nm, respectively. The DNA‐histone complex was measured with Cell Death Detection ELISA^PLUS^ (Roche Diagnostics GmbH), based on the principle of sandwich enzyme immunoassay, in which mouse monoclonal antibodies directly binding to DNA and histones were used for this assay. These processes enable the quantification of nucleosomes in the cytoplasmic fraction of cell lysates. The number of nucleosomes in the immunocomplex formed by the anti‐histone antibody is photometrically determined using a VersaMax^TM^ Microplate Reader (Molecular Devices). The results were expressed by absorbance difference ([A_405 nm_−A_490 nm_] × 1000 mU). Quality control for the assays was conducted according to their manufacturer's instructions, and they were routinely calibrated according to the manufacturer's recommendations.

### 2.3 Statistical analyses

Statistical analyses were performed using MedCalc for Windows, version 18.10 (MedCalc Software). A *P*‐value <.05 was considered to be significant. All data were expressed as numbers with percentages or mean ± standard deviation. To compare continuous variables with the control group, the independent *t* test was used for variables with parametric distribution, and the Mann‐Whitney test was used for variables with nonparametric distribution. Analyses of categorical variables were performed by the chi‐square test. Box and whisker plots were used to show the distributions of dsDNA concentration values for ACS, AIS, and control groups. To investigate significant variables of ACS and AIS, the multivariable Cox regression analyses using the enter method were performed on variables with a *P*‐value of <.20 in the univariable analyses. The area under the curve (AUC) values were obtained by using the receiver operating characteristic (ROC) curves for the variables showing statistically significant results. During the follow‐up period, occurrences of MACEs were analyzed using the Kaplan‐Meier method.

## 3 RESULTS

### 3.1 Baseline characteristics of the study population

At baseline, there were no statistically significant differences between the ACS and the control groups, and between the AIS and the control groups for the following variables: age, gender, underlying diseases (diabetes mellitus [DM], hypertension, and dyslipidemia), social histories (alcohol intake and current smoking), and laboratory data (PT and aPTT). Neutrophil count, C‐reactive protein, troponin I (TnI), and creatinine kinase MB (CK‐MB) levels were significantly higher in both ACS and AIS groups than in the control group, and platelet count in both ACS and AIS groups was significantly lower than in the control group. The baseline characteristics of the study population are summarized in Table 1.

**Table 1:** Baseline characteristics of the study population

Variables	ACS (N = 37)	AIS (N = 58)	Control (N = 25)	*P*	
				ACS vs. control	AIS vs. control
Demographic characteristics					
Age (y), mean ± SD	67.1 ± 10.1	68.2 ± 11.3	66.4 ± 7.1	.754^a^	.374^a^
Male, no. (%)	27 (73.0)	34 (58.6)	15 (60.0)	.288	.907
Underlying diseases					
Diabetes mellitus, no. (%)	15 (40.5)	21 (36.2)	7 (28.0)	.315	.471
Hypertension, no. (%)	23 (62.2)	40 (69.0)	14 (56.0)	.630	.259
Dyslipidemia, no. (%)	12 (32.4)	12 (20.7)	7 (28.0)	.713	.470
Social history					
Alcohol intake, no. (%)	8 (21.6)	23 (39.7)	8 (32.0)	.364	.511
Current smoking, no. (%)	15 (40.5)	16 (27.6)	8 (32.0)	.498	.686
Laboratory data					
Neutrophil (×10^9^/L)	4.46 ± 1.58	5.01 ± 1.79	2.63 ± 0.95	**<.001** a	**<.001** b
Platelet (×10^9^/L)	203.7 ± 49.6	206.2 ± 55.8	247.6 ± 53.2	**.002** b	**.005** b
CRP (mg/dL)	0.18 ± 0.13	0.66 ± 1.22	0.10 ± 0.11	**.001** b	**<.001** b
PT (s)	12.2 ± 0.9	12.4 ± 0.9	12.2 ± 0.6	.631^b^	.379^b^
aPTT (s)	24.6 ± 2.9	24.0 ± 2.0	24.2 ± 2.3	.901^a^	.689^a^
TnI (pg/mL)	299.4 ± 638.8	26.3 ± 93.0	3.0 ± 4.4	**<.001** b	**<.001** b
CK‐MB (U/L)	16.0 ± 6.2	19.2 ± 15.9	13.4 ± 7.0	**.024** b	**.001** b

Note

Continuous data are presented as mean ± standard deviation, and categorical data are presented as numbers with percentages in round brackets.

Abbreviations: ACS, acute coronary syndrome; AIS, acute ischemic stroke; aPTT, activated partial thromboplastin time; CK‐MB, creatinine kinase MB; CRP, C‐reactive protein; PT, prothrombin time; TnI, troponin I; WBC, white blood cell.

a Comparisons by independent *t* test.

b Comparisons by Mann‐Whitney test.

*P*‐values lower than 0.05 were considered to be significant and they were shown in bold.

### 3.2 Comparison of the circulating levels of NETs between the ACS, AIS, and control group

**Figure 1:**
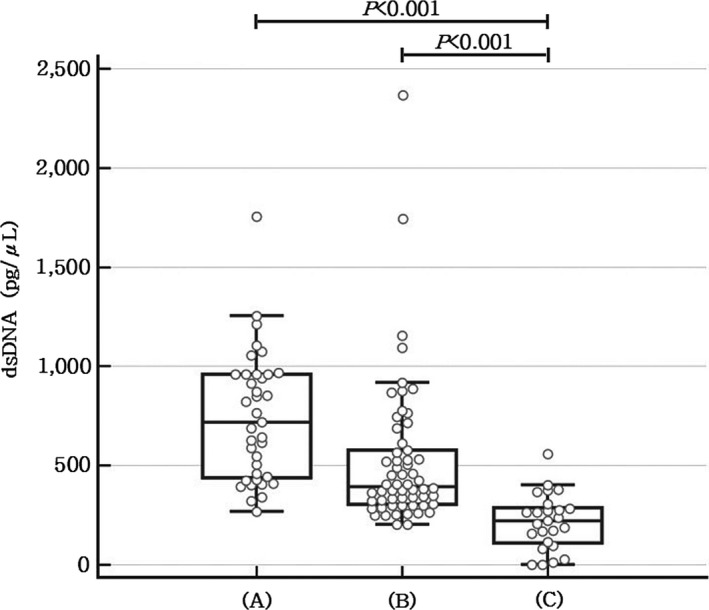
The distributions of dsDNA concentration values with box and whisker plots for (A) acute coronary syndrome, (B) acute ischemic stroke, and (C) healthy control groups

The circulating levels of NETs evaluated via measuring dsDNA concentrations were significantly higher in the ACS (743.28 ± 323.10 pg/μL) and AIS (524.22 ± 370.06 pg/μL) groups than in the control group (216.48 ± 140.43 pg/μL), both *P* < .001. Distributions of dsDNA concentrations for each group are depicted in Figure 1. On the Kolmogorov‐Smirnov test, the dsDNA concentrations of the ACS group showed a parametric distribution, but those of the AIS group did not. The measured values of DNA‐histone complex in the ACS (19.73 ± 34.19 mU) and AIS (13.71 ± 16.96 mU) groups were not statistically different from those in the control group (14.32 ± 8.86 mU), *P* = .364 and *P* = .830, respectively, and data not shown.

### 3.3 Univariable and multivariable analyses for the ACS and AIS groups

For the Cox regression analyses, the cutoff values were set as the optimal points using Youden's index method for each variable. The univariable analyses showed statistically significant results for all variables except CK‐MB level in the ACS group (*P* = .081). In the multivariable analyses for variables with *P*‐values <.20 in the univariable analyses, TnI level, and dsDNA concentration were statistically significant risk factors in the ACS group (*P* = .046 with an odds ratio of 27.49 [95% CI, 1.06‐714.90] and *P* = .015 with an odds ratio of 42.94 [95% CI, 2.11‐873.94], respectively). In the AIS group, only dsDNA concentration was a statistically significant risk factor (*P* = .002 with an odds ratio of 13.31 [95% CI, 2.51‐70.54]). The results of the univariable and multivariable Cox regression analyses are shown in Table 2.

**Table 2:** Results of the Cox regression analyses for dsDNA concentration and other variables in ACS and AIS patients

Variables	Univariate			Multivariate		
	OR	95% CI	*P*	OR	95% CI	*P*
ACS						
Neutrophil						
(>3.4074 × 10^9^/L vs. ≤3.4074 × 10^9^/L)	31.43	7.30‐135.35	**<.001**	18.18	0.68‐485.13	.084
Platelets						
(<205 × 10^9^/L vs. ≥205 × 10^9^/L)	6.18	1.77‐21.55	**.004**	6.45	0.32‐130.66	.225
CRP						
(>0.09 mg/dL vs. ≤0.09 mg/dL)	5.74	1.89‐17.41	**.002**	3.23	0.22‐47.06	.391
TnI						
(>5.9 pg/mL vs. ≤5.9 pg/mL)	19.80	4.85‐80.91	**<.001**	27.49	1.06‐714.90	**.046**
CK‐MB						
(>12.0 U/L vs. ≤12.0 U/L)	2.56	0.89‐7.35	**.081**	1.07	0.07‐15.61	.958
dsDNA						
(>400.69 pg/μL vs. ≤400.69 pg/μL)	94.88	16.02‐562.00	**<.001**	42.94	2.11‐873.94	**.015**
AIS						
Neutrophil						
(>3.9013 × 10^9^/L vs. ≤3.9013 × 10^9^/L)	19.25	5.06‐73.27	**<.001**	4.62	0.63‐33.90	.132
Platelets						
(<208 × 10^9^/L vs. ≥208 × 10^9^/L)	4.90	1.50‐16.06	**.009**	3.12	0.40‐24.35	.277
CRP						
(>0.12 mg/dL vs. ≤0.12 mg/dL)	9.08	3.05‐27.00	**<.001**	2.57	0.53‐15.41	.301
TnI						
(>3.7 pg/mL vs. ≤3.7 pg/mL)	9.98	3.01‐33.07	**<.001**	7.48	0.97‐57.68	.054
CK‐MB						
(>14.0 U/L vs. ≤14.0 U/L)	7.04	2.41‐20.58	**<.001**	5.96	0.98‐36.31	.053
dsDNA						
(>281.28 pg/μL vs. ≤281.28 pg/μL)	19.79	6.06‐64.60	**<.001**	13.31	2.51‐70.54	**.002**

Abbreviations: ACS, acute coronary syndrome; AIS, acute ischemic stroke; CI, confidence interval; CK‐MB, creatinine kinase MB; CRP, C‐reactive protein; dsDNA, double‐stranded DNA; OR, odds ratio; TnI, troponin I.

*P*‐values lower than 0.05 were considered to be significant and they were shown in bold.

### 3.4 ROC curve analyses for the ACS and AIS groups

**Figure 2:**
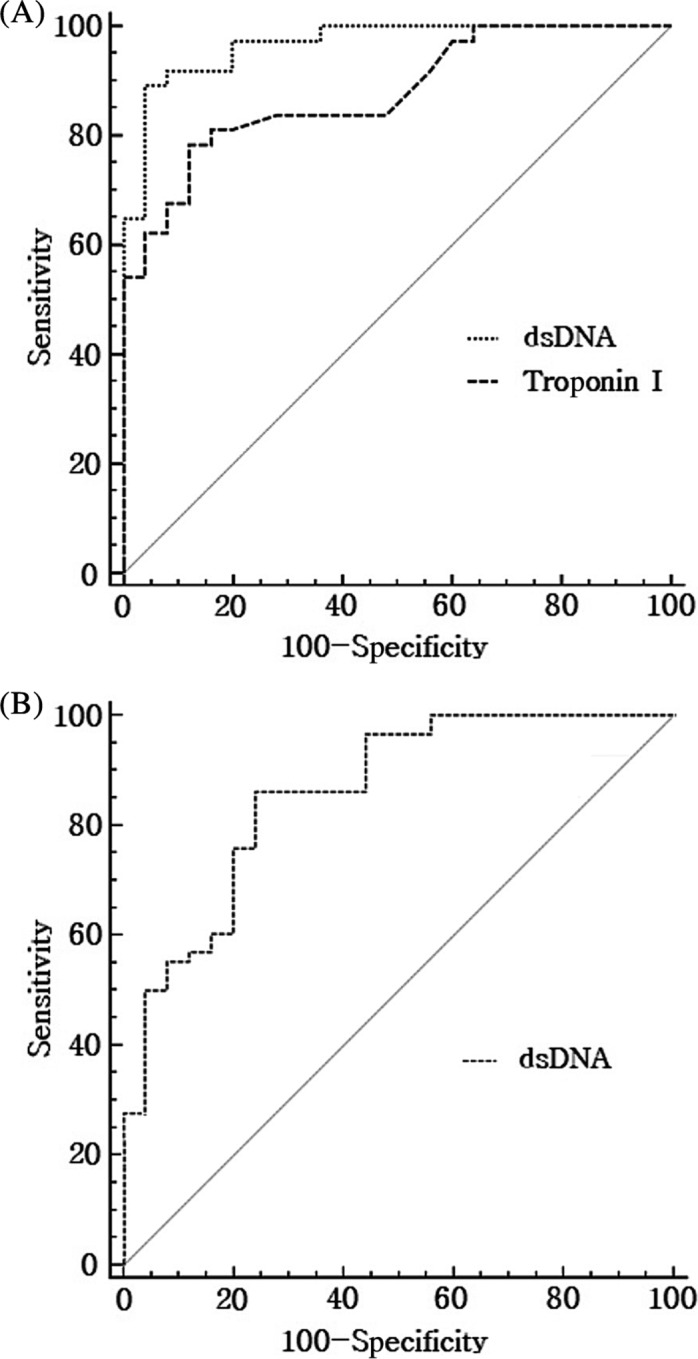
Receiver operating characteristic (ROC) curves (A) for troponin I and dsDNA in acute coronary syndrome and (B) for dsDNA in acute ischemic stroke

The ROC curve analyses for the ACS and AIS groups were performed on the variables showing significant results in the Cox regression analyses (Figure 2). In the AIS group, ROC curve analysis was performed only on dsDNA concentration, which showed a statistically significant result in the previous multivariable analyses. In the ACS group, the AUC values for TnI level and dsDNA concentration were 0.878 and 0.968, respectively, although there was no statistically significant difference in comparison of the two ROC curves (*P* = .060). The AUC value for dsDNA concentration in the AIS group was 0.859 (Table 3). For dsDNA concentrations in the ACS and AIS groups, cutoff values representing an optimal sensitivity/specificity pair were calculated as 400.69 pg/μL (sensitivity of 89.2% and specificity of 96.0%) and 281.28 pg/μL (sensitivity of 86.2% and specificity of 76.0%), respectively.

**Table 3:** AUC and *P*‐value of each variable by the ROC curve analyses

Variables	AUC	SE	95% CI	*P*
ACS				
Troponin I	0.878	0.0422	0.770‐0.947	<.001
dsDNA	0.968	0.0190	0.888‐0.996	<.001
AIS				
dsDNA	0.859	0.0454	0.765‐0.925	<.001

Note

The SE value of ROC curve was obtained by the method of Delong et al (1988), and the 95% CI of AUC was calculated by binominal exact method.

Abbreviations: ACS, acute coronary syndrome; AIS, acute ischemic stroke; AUC, area under the curve; CI, confidence interval; dsDNA, double‐stranded DNA; ROC, receiver operating characteristic; SE, standard error.

### 3.5 Time‐to‐event analyses for the occurrence of MACEs

**Figure 3:**
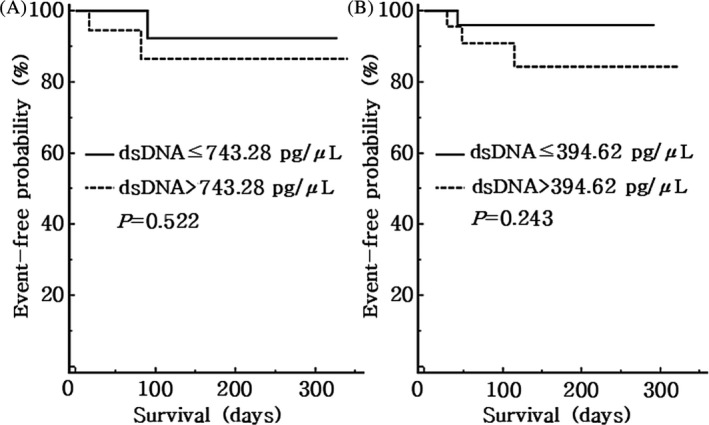
Kaplan‐Meier event‐free cumulative curves for dsDNA concentrations in patients with (A) acute coronary syndrome (ACS) and (B) acute ischemic stroke (AIS)

For time‐to‐event analyses, using the Kaplan‐Meier method, each patient group was divided into two subgroups according to their mean and median values of the dsDNA concentrations: 743.28 pg/μL for the ACS group and 394.62 pg/μL for the AIS group, respectively. Results of the time‐to‐event analyses showed that more MACEs occurred in the subgroups with higher dsDNA concentrations than each value (Table 4), but there were no statistically significant differences between the subgroups of each group, the ACS and AIS (*P* = .522 and *P* = .243, respectively). The Kaplan‐Meier event‐free cumulative curves for dsDNA concentrations in patients with ACS and AIS are depicted in Figure 3. When comparing the event‐free probability based on the median values of TnI, there were also no statistically significant differences in the ACS and AIS groups (*P* = .059 and *P* = .171, respectively, and data not shown).

**Table 4:** Characteristics of patients who suffered from additional MACEs during the follow‐up period

Age (y)	Sex	Initial diagnosis	dsDNA (pg/μL)	Group	MACE	Follow‐up period (d)
80	M	UA	1072.49^a^	ACS	Myocardial infarction (UA)	17
57	M	NSTEMI	1103.30^a^	ACS	Myocardial infarction (NSTEMI)	82
72	M	NSTEMI	404.61	ACS	Myocardial infarction (NSTEMI)	90
83	F	Rt. MCA infarction	1092.61^a^	AIS	Ischemic stroke (Rt. MCA infarction)	29
57	M	Rt. CR‐BG infarction	371.76	AIS	Ischemic stroke (Rt. BG infarction)	42
55	F	Multiple cerebral infarction	2360.51^a^	AIS	Ischemic stroke (Multiple cerebral infarction)	48
62	M	Lt. MCA infarction	871.93^a^	AIS	Ischemic stroke (Rt. ICA infarction)	114

Abbreviations: ACS, acute coronary syndrome; AIS, acute ischemic stroke; BG, basal ganglia; CR, corona radiata; dsDNA, double‐stranded DNA; ICA, internal carotid artery; MACEs, major adverse cardiac events; MCA, middle cerebral artery; NSTEMI, non‐ST elevation myocardial infarction; UA, unstable angina.

a Higher dsDNA concentrations than the mean value of the ACS group; 743.28 pg/μL and the median value of the AIS group; 394.62 pg/μL.

## 4 DISCUSSION

In the present study, the circulating levels of NETs were evaluated by measuring dsDNA concentrations in patients with ACS and AIS. After the Cox regression analyses were performed to determine significant variables in ACS and AIS, the ROC curve analyses were carried out to confirm whether dsDNA concentration could be a useful marker at the initial stages of ACS and AIS. We also investigated occurrences of MACEs during the follow‐up period to check whether the elevated dsDNA concentration would be associated with poor prognosis in patients with ACS and AIS.

The increased level of NETs has been shown to be triggered by certain clinical conditions such as infection, sepsis, autoimmune diseases, atherosclerosis, deep vein thrombosis, and thrombotic microangiopathy.^15^, ^16^, ^17^, ^18^, ^19^ The role of neutrophils in thrombus formation and amplification has been illustrated by several previous studies, and the findings that neutrophils produce NETs consisting of chromatin networks and cytoplasmic proteins have provided a new perspective on the mechanisms involved in thrombosis.^10^, ^20^, ^21^ ACS is a term encompassing clinical disorders of myocardial ischemia or infarction, and it has been demonstrated that inflammatory responses involving neutrophil activation play a major role in atherosclerotic changes and progression.^22^, ^23^, ^24^, ^25^ According to a recent study, circulating extracellular DNA levels in AIS patients are associated with innate immune system activation.^26^ Though clear mechanisms are yet to be explored, these processes could lead to the elevated level of NETs in the ACS and AIS groups in this study. In this respect, it is necessary to examine the possibility that the increased level of NETs is associated with the pathogenesis of ACS and AIS as one of many damage‐associated molecular patterns.

Though the presence of DM was not considered as a confounding factor in this study, a previous in vitro study discovered that production of NETs from isolated neutrophils was promoted by high glucose levels, and especially, type 2 DM patients with elevated glycated hemoglobin (HbA1c) had high levels of nucleosomes, neutrophil elastase, and other components of NETs.^27^ Meanwhile, dyslipidemia could injure endothelial cells and induce neutrophilia, which is eventually associated with the promotion of lipid deposition and atherosclerotic plaque burden.^28^ In addition, dyslipidemia could promote serum levels of C‐X‐C motif ligand (CXCL)‐1, which enhances neutrophil mobilization.^29^ In the present study, there was no statistical difference in the prevalence of DM, hypertension, and dyslipidemia of the ACS, AIS, and control groups, and this is probably because the control group was selected, in part, according to age. TnI and CK‐MB were higher in AIS patients than those in the control group. In interpreting the cardiac markers, such as TnI and CK‐MB in AIS patients, there are various hypotheses about how cerebrovascular events directly endanger the heart, but there is no convincing mechanistic explanation as of yet.^30^, ^31^, ^32^, ^33^, ^34^, ^35^

Based on the results of this study, it was confirmed that the circulating level of NETs increased in patients with ACS or AIS at initial presentation. In particular, the dsDNA concentrations in the ACS group were significantly higher, not only to those in the control group but also to those in the AIS group. This is, perhaps, due to the lack of consideration of disease severity in the AIS group at the time of admission. According to the previous study of Vallés et al, 2017, a positive correlation exists between NETs level and the National Institutes of Health Stroke Scale (NIHSS) score, reflecting stroke severity, and patients with a higher NIHSS score had a higher NETs level (*P* < .001).^36^

Interestingly, the results of the ROC curve analyses for the ACS group showed that the dsDNA concentration would be an equivalent marker to TnI. Additionally, the ROC curve analysis for patients with UA, having suggestive symptoms of cardiac ischemia without elevated TnI levels, showed a slightly higher AUC value of 0.973 (*P* < .001, data not shown). These results suggest that measuring the dsDNA concentration could be a more useful marker than TnI for the initial diagnosis of UA.

During a relatively short period, we investigated the occurrence of MACEs in the ACS and AIS groups. Although there was no statistically significant difference between subgroups, there was a tendency for more MACEs to occur in the subgroups with higher dsDNA concentrations. There have been few prospective studies researching the predictability of the circulating levels of NETs for the occurrence of MACEs. Moreover, subjects of the previous studies were limited to patients with coronary atherosclerosis, STEMI, and hemodialysis.^17^, ^37^, ^38^ To the best of our knowledge, this is the first study to investigate the occurrence of MACEs with the circulating levels of NETs in the ACS and AIS groups together.

This study has some methodological limitations. The first is a relatively small number of subjects; all of them were enrolled from a single hospital. Future research on this topic should aim to include a larger study population. Furthermore, the dsDNA concentrations were measured using leftover samples of the patients from initial presentation, but strictly speaking, the time after the actual onset of their diseases might be different for each patient. It would be beneficial to study the changes in dsDNA concentrations according to time course or disease progression, intra‐individually.

As noted above, the lack of consideration given to disease severity in the AIS group at the time of admission could also be a limitation. Under consideration of the disease etiology with a larger study population, it would be meaningful to compare the dsDNA concentrations with various indicators of disease severity, such as the NIHSS and modified Rankin Scale.

Furthermore, a long‐term follow‐up period would be necessary to obtain statistically significant results in predictions of disease prognoses. In this study, we simply divided the subgroups by their mean and median values according to the number of participants. If the occurrence of MACEs were analyzed by dividing the patient groups into more dummy variables, based on the dsDNA concentration, the results may be more explicitly detailed. As well, analysis of the changes in circulating levels of NETs during the follow‐up period may be more meaningful.

In the first report on NETs, immunofluorescence staining of histological sections was performed to demonstrate the presence of extracellular fibrous material containing NET components.^10^ Since then, previous studies have used various methods for evaluating the suitability of NETs as biomarkers in the aforementioned clinical conditions, in which the main analytical targets were cell‐free DNA, histones, and other components of NETs, such as neutrophil elastase or myeloperoxidase in plasma.^39^ Therefore, it is necessary to find a marker that could be clinically useful for disease diagnosis, early prediction of severity, and prognosis estimation. It is important to establish a standardized or practically recommended analyzing method, which uses a straightforward process along with its cost‐effectiveness in a clinical laboratory. Much to our regret, the measured values of DNA‐histone complex did not show any statistically significant results in this study, which is probably because of the lack of standardized sample preparation steps and a well‐organized analysis protocol suitable for the purpose. Further studies should be conducted in order to provide a realized benefit to patients using the discovery of NETs.

On the other hand, there have been recent studies on chronic neurological diseases using cell‐free DNA in cerebrospinal fluid (CSF) samples.^40^, ^41^, ^42^ There was a study that analyzed serial nuclear and mitochondrial DNA levels in plasma and CSF for patients with subarachnoid hemorrhage.^43^ It would be worthwhile to investigate whether the levels of NETs in CSF samples could be a superior marker for the diagnosis of AIS.

In summary, this study demonstrated that measuring the circulating levels of NETs has diagnostic power equivalent to TnI in ACS patients at initial presentation. Similarly, NETs analyses in patients with AIS showed potential for their use as novel circulating markers for the initial diagnosis of AIS.

## ETHICAL APPROVAL

This study was approved by the institutional review board of Dong‐A University Hospital. Written consents from the patients were waived because it was a study using residual samples without any additional blood collection.
